# Three Dimensional Neuronal Cell Cultures More Accurately Model Voltage Gated Calcium Channel Functionality in Freshly Dissected Nerve Tissue

**DOI:** 10.1371/journal.pone.0045074

**Published:** 2012-09-25

**Authors:** Yinzhi Lai, Ke Cheng, William Kisaalita

**Affiliations:** Cellular Bioengineering Laboratory, College of Engineering, University of Georgia, Athens, Georgia, United States of America; Georgia State University, United States of America

## Abstract

It has been demonstrated that neuronal cells cultured on traditional flat surfaces may exhibit exaggerated voltage gated calcium channel (VGCC) functionality. To gain a better understanding of this phenomenon, primary neuronal cells harvested from mice superior cervical ganglion (SCG) were cultured on two dimensional (2D) flat surfaces and in three dimensional (3D) synthetic poly-L-lactic acid (PLLA) and polystyrene (PS) polymer scaffolds. These 2D- and 3D-cultured cells were compared to cells in freshly dissected SCG tissues, with respect to intracellular calcium increase in response to high K^+^ depolarization. The calcium increases were identical for 3D-cultured and freshly dissected, but significantly higher for 2D-cultured cells. This finding established the physiological relevance of 3D-cultured cells. To shed light on the mechanism behind the exaggerated 2D-cultured cells’ functionality, transcriptase expression and related membrane protein distributions (caveolin-1) were obtained. Our results support the view that exaggerated VGCC functionality from 2D cultured SCG cells is possibly due to differences in membrane architecture, characterized by uniquely organized caveolar lipid rafts. The practical implication of use of 3D-cultured cells in preclinical drug discovery studies is that such platforms would be more effective in eliminating false positive hits and as such improve the overall yield from screening campaigns.

## Introduction

A key goal in cell-based assay technology is to achieve cellular responses to external stimuli that are physiologically relevant to what happens *in vivo* as closely as possible. However, many whole-cell-based assays in use today rely on flat, two-dimensional (2D) glass or plastic substrates that may not produce results characteristic of *in vivo* conditions. Three dimensional (3D) substrates or scaffolds provide cells with *in vivo*-like topographical cues and thus enable cells to differentiate into specific phenotype and maintain specific functions that are usually impossible under 2D cell culture conditions [1], [2], [3]. Thus 3D cell-based assay systems are desirable in preclinical drug discovery applications.

Various approaches and materials have been studied for creating three-dimensionality. Among them are microgravity bioreactors [4], [5], natural polymers especially collagen hydrogels [6], [7], [8], photopolymerized hydrogels [9], synthetic polymer scaffolds [10], [11], self-assembling peptide scaffolds [12], and micro/nano patterned substrates [13].

Synthetic polymer scaffolds offer several comparative advantages. First, in comparison to microgravity bioreactors and patterned substrates, polymer scaffolds are easier and faster to prepare and can be scaled down for incorporation into high throughput screening (HTS) systems. Second, in comparison to most hydrogels, polymer scaffolds offer less resistance to diffusion of nutrients and wastes to and from cells deeper in scaffold [7]. Third, in comparison to nanoscale pores and fibers associated with self-assembling peptide scaffolds, microscale pores in polymer scaffolds are large enough to host 3D multi-cellular aggregates or microtissues [14].

We have previously demonstrated that polymer based scaffolds can be integrated into HTS compatible platform for whole-cell-based assays [15], [16], [17]. Neural stem cells cultured in such polymer based scaffolds modeled *in vivo* surrogate (neural spheres) better than 2D cells in terms of morphology, cellular growth profile, gene expression and voltage gated calcium channel (VGCC) function [15] – VGCC are emerging drug targets [18], [19]. We extend our previous study herein and show that primary neuronal cells from mouse superior cervical ganglion (SCG) cultured in 3D polymer scaffolds model freshly dissected tissue with respect to VGCC function. Furthermore, our results suggest a role for membrane architecture in higher VGCC signal, in response to high K^+^ depolarization observed in 2D cultures. Overall, our results support the view that 2D/3D cultured cells’ plasma membrane architecture differences are worthy of further exploration in efforts to better understand 3D cultured cells’ better emulation of the *in vivo* function.

## Materials and Methods

### Ethics Statement

All the animals received the standard care in compliance with the Animal Welfare Act and the recommendation in the Guide for the Care and Use of Laboratory Animals of the National Institutes of Health. The protocol was approved by the Committee on the Ethics of Animal Experiments of the University of Georgia and was under the University of Georgia Animal Usage Proposal (Permit Number: A2009 3-048).

### Scaffold Fabrication

3D Scaffolds were fabricated by the gaseous salt leaching method [20] and have been previously described in detail [15]. Briefly, a highly viscous polymer solution was prepared by dissolving polymer particles in chloroform. Ammonium bicarbonate salt particles were added to the polymer solution and mixed thoroughly. Sieved ammonium bicarbonate particles in the range of 40–60 µm in diameter were used. Salt particles in this size range generated pores with an average diameter range of 60–100 µm. The salt: polymer ratio of 20∶1 was chosen to achieve the optimal balance between light transmission and mechanical strength. The paste mixture of polymer/salt/solvent was cast onto a glass Petri dish. After chloroform was partially evaporated under atmospheric pressure, the semi-solidified samples were immersed into boiling water until no gas bubbles were generated. The samples were vacuum-dried for 24 h and kept in desiccators until use. Before cell plating, the scaffolds were pre-wetted and sterilized in 70% ethanol under UV light, over night, and then rinsed with PBS three times. The scaffolds were coated with collagen by incubating in 0.1 mg/ml collagen solution for 2 h, followed by air drying at room temperature.

### Scanning Electron Microscopy (SEM)

Cells in scaffolds were fixed with 2% glutaraldehyde in 0.1 M sodium cacodylate buffer (pH 7.2) for 1 h and then rinsed in cacodylate buffer three times (15 min each). This was followed by post-fixing with 1% OsO_4_ in 0.1 M sodium cacodylate buffer for 1 h and rinsing in cacodylate buffer three times (5 min each). The samples were then dehydrated in 35, 50, 70, 80, 95 and 100% ethanol successively for 10 min each and dried in a SAMDRI-780A critical point drier (Tousimis Research Corporation, MD). Scaffolds were sputter-coated with gold for 60 s to achieve a thickness of about 15.3 nm. SEM images were captured with LEO 982 scanning electron microscope (LEO Electronenmikroskopie GmbH Korporation, Germany) with an acceleration voltage of 4 kV. A similar protocol was followed for scaffold samples without cells, with the exception that the preparation started with sputter coating.

### SCG Cell Harvesting and Plating

Neonatal mice (CD1) were used as the sources of SCG nerve cells. A mice SCG dissection protocol described elsewhere [21] was followed. All the animals received the standard care in compliance with the Animal Welfare Act and the recommendation in the Guide for the Care and Use of Laboratory Animals of the National Institutes of Health. The protocol was approved by the Committee on the Ethics of Animal Experiments of the University of Georgia and was under the University of Georgia Animal Usage Proposal (Permit Number: A2009 3-048). After dissection, the ganglia were enzymatically digested in 1 mg/ml type IA collagenase for 1 hour. After gentle mechanical disruption with a Pasteur pipette, dissociated cells and cell chunks were plated into glass bottom Petri dishes (MatTek, MD) and polymer scaffolds, both of which were coated with 0.1 mg/ml type I collagen. On the average, cells from two ganglia were plated to one dish or scaffold. Cells were maintained in Eagle’s Minimum Essential Medium supplemented with 2 mM L-Glutamine, 1 mM Sodium Bicarbonate, 10% FBS, and 50 ng/ml NGF. The cells were incubated at 37°C in a 10% CO_2_ humidified atmosphere. To prepare intact SCG tissue samples for staining, the outer sheath covering the freshly dissected ganglia was broken with fine forceps.

### Live Cell Imaging and Morphological Studies

SCG cell morphology was observed by calcein acetoxymethyl ester (AM) (Biotium, Hayward, CA) staining. The non-fluorescent cell-permeant calcein AM is converted to non-permeable intensely fluorescent calcein by the intracellular esterase thus are widely utilized to stain living cells and their extensions [22]. Cells were washed with 2 mL PBS, three to five times (5 min each) then covered with sufficient amount of 2-µM calcein AM in PBS. After 30 min incubation at room temperature, the staining solution was replaced with fresh PBS, followed by another 30 min incubation complete esterification of the intracellular calcein AM. Confocal images were obtained by 488 nm argon laser excitation and recorded through a 515 nm long Pass filter.

### VGCC Functionality Characterization

Calcium imaging experiments were performed after 2-day and 7-day incubation for both the 2D and 3D cultured cell samples. A typical mouse SCG is fusiform in shape with center diameter less than 1 mm, which allows confocal imaging of the intact tissue. To best represent the *in vivo* condition, intact SCG tissues were stained soon after the dissection, followed by the calcium imaging. The time between dissection and recording was approximately one hour. Intracellular calcium dynamics were recorded using the membrane permeable dye Calcium Green-1 AM coupled with confocal laser scanning microscopy. 2D cultured cells on the Petri dish were washed twice with HEPES buffered saline (HBS) and loaded with 5 µM dye in 1 ml of HBS containing 3% FBS and 0.02% Pluronic F-127. The Petri dishes were incubated at 37°C for 30 min. After dye loading, cells were rinsed with HBS twice and returned to the incubator for another 30 min to allow complete dye de-esterification. A similar protocol was followed for 3D and intact SCG tissue samples. However, the dye concentration was increased to 10 µM to facilitate dye loading. Both the scaffold and intact SCG tissue were held in place by placing a small coverslip on top during the calcium imaging. Calcium Green-1 was excited with 488 nm argon laser and the fluorescence intensity was recorded through a 515 nm long Pass filter. Images were taken every 3 seconds. Cells were depolarized by adding 100 µl of high potassium buffer to a final concentration of 50 mM K^+^ while imaging. The intracellular calcium dynamics were reflected by changes in intracellular Calcium Green-1 fluorescence intensity.

### Microarray Gene Expression Analysis

Total RNA was isolated from all samples using Qiagen RNeasy Kits (Qiagen, Valencia, CA) according to the manufacturer’s standard protocol. The quantity of mRNA isolated from each sample was determined using the adsorption of each solution at 260 nm and 280 nm. The purity of each sample was monitored using the A260/A280 ratio. A ratio of 1.8–2.1 was considered a “clean” sample and could be used in microarray experiments. Samples were kept on dry ice and sent to the Affymetrix Core Facility at the Medical College of Georgia (MCG) for Mouse Genome 430 2.0 array Expression Analysis (Affymetrix, Santa Clara, CA). The raw data has been deposited in MIAME compliant GEO database (Accession No. GSE30498). The expression value of each gene was obtained by Expression Console (Affymetrix) with the Robust Multichip Average (RMA) algorithm which consists of three steps: a background adjustment, quantile normalization and finally summarization. RMA was chosen because it is one of the most widely used algorithms and has been shown to be more sensitive than other methods. Genes within the scope of this study were manually picked. Student’s t tests and ANOVA tests were carried out to compare the expression differences between 2D, 3D and SCG tissue samples.

### Immuno-fluorescence Staining

Rabbit polyclonal Anti-Calcium channel L type DHPR alpha 2 subunit antibody (ab80990) and sheep polyclonal caveolin-1 antibody (ab81397) were purchased from Abcam (Cambridge, MA). Focal Adhesion Staining Kit was purchased from Millipore (Billerica, MA). Secondary antibodies (Alexa Fluor 488 chicken anti-rabbit and Alexa Fluor 488 donkey anti-sheep) were purchased from Invitrogen (Carlsbad, CA). Normal chicken serum was purchased from Zymed Laboratories, CA. For immuno-fluorescence staining, cells were rinsed once with phosphate-buffered saline (PBS), fixed with 4% paraformaldehye in PBS (30 min), washed with PBS, treated with 0.5% Triton X-100 (5 min) for membrane permeablization. To double stain L-type VGCC and caveolin-1, cells were washed with PBS, blocked with normal chicken serum (2% diluted with PBS/0.3% Tween-20) for 30 min at room temperature, washed with PBS (3 times, 5 min per wash), incubated overnight with primary antibodies (1∶100), washed with PBS (3 times, 5 min per wash) followed by incubation for 1 h with the secondary antibodies (1∶500), washed with PBS (3 times, 5 min per wash), and then loaded with DAPI (1∶5000) for 5 min at room temperature. Confocal imaging was performed with a Leica TCS SP2 microscope. Routine negative controls for staining were performed.

### Statistical Analysis

Neurite length, soma section area, soma section roundness and calcium response magnitude values were expressed as mean ± standard deviation (S.D.). The unpaired Student’s t-test was used to compare the means of two samples. The *p* values are indicated in the text and decisions regarding significant difference were based on level of 0.05.

## Results

### SCG Cell-scaffold Interaction

SCG cells were found ideal for this study because of the ease with which freshly dissected ganglion tissue can be obtained to serve as a more realistic *in vivo* surrogate as opposed to neural spheres used in our previous study [15]. Also, we used the CD1 mouse strain as the source of SCG cells and tissue because this strain has been extensively used in toxicological and functional studies as an acceptable model for human medicine applications.

Mouse (SCG) cells were harvested and cultured on the 3D polymer scaffolds (made from poly-l-lactic-acid (PLLA) or polystyrene (PS)) and 2D substrates (glass or polystyrene) [11]. All 3D and 2D substrates were coated with 0.1 mg/ml Type I collagen from rat tail to rule out any differences from materials. There were no statistically significant differences between the architecture of scaffolds generated from PLLA and PS and as such the scaffolds had similar impact on cells (data not shown). However, while PLLA scaffolds are suitable for *in vivo* studies that require scaffold biodegradation, PS scaffolds practically do not biodegrade and are only suitable for *in vitro* studies where degradation is not a concern. In this study, porous polymer scaffolds with equivalent average pore sizes of 60–100 µm in diameter were fabricated. This pore size range was empirically found to be ideal for mouse SCG cells, which are approximately 10 µm in diameter. Cell intrusion (penetration deep into pores) requires larger pores and pore openings, however, if too large, the pore would have no difference from the 2D situation and would make cell-matrix interaction identical to that found on flat surfaces [23]. The porosity of resulting scaffolds ranged between 88.4% and 95.6%, and the pores were inter-connected so that cells can travel between pores through channels and/or form neurites connections between two adjacent cell aggregates. As discussed in Cheng et al. [23], we used a polymer to salt ratio of 1∶20 to achieve the optimal light transmittance (80% in wet condition) while maintaining adequate mechanical strength, which is higher than the maximum possible force a typical fluid transfer workstation (e.g. FLEXstation, Molecular Devices, Sunnyvale, CA) could generate (0.11 mN). The detailed physical characteristics of those polymer scaffolds have been reported previously by Cheng et al. [23].

**Figure 1:**
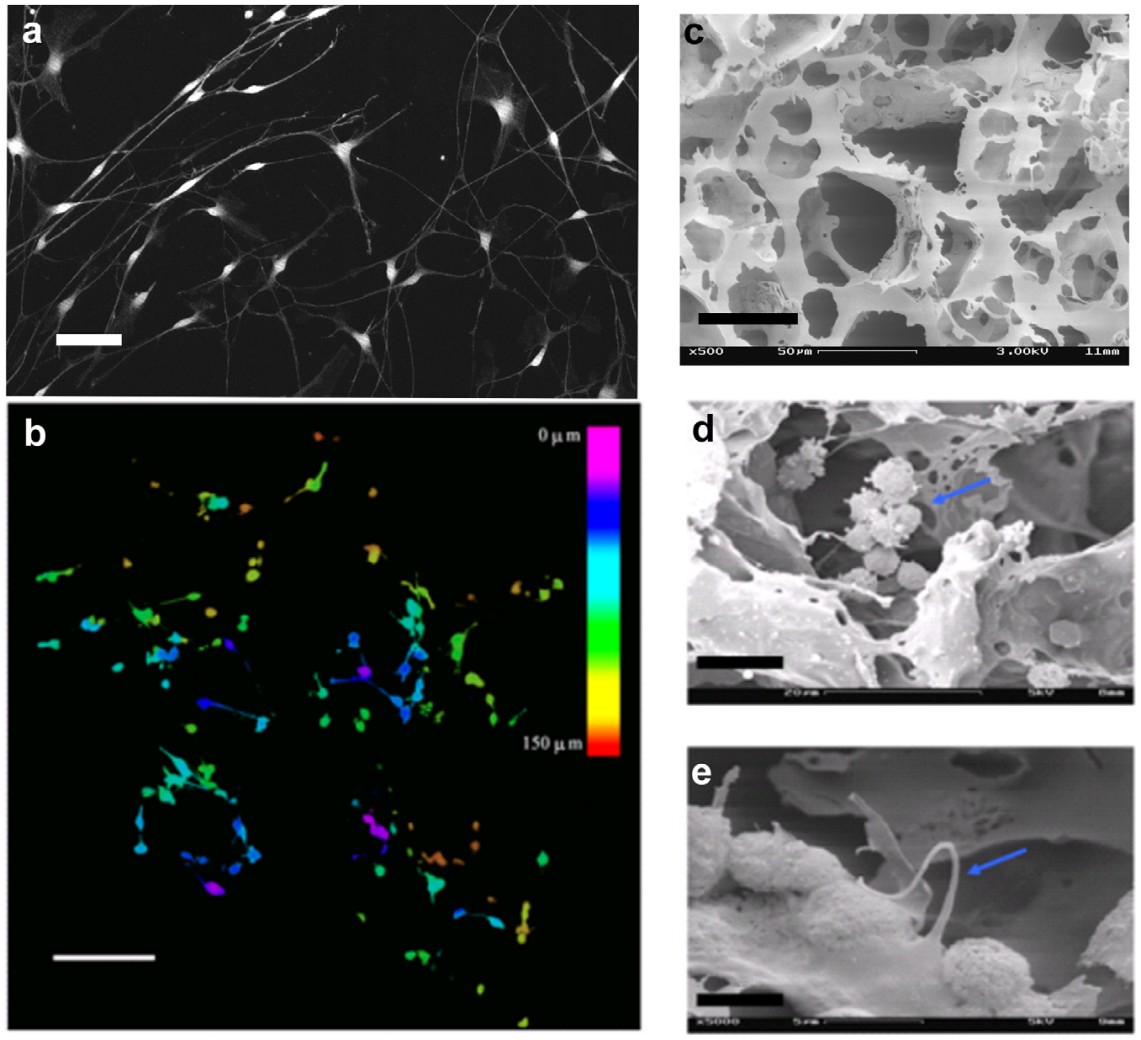
Polymer (PLLA) scaffolds seeded with SCG cells. (a) Confocal image with calcein stained SCG cells cultured on flat surfaces. (b) Confocal depth projection micrograph of a 20∶1 PLLA polymer scaffold with 60–100 µm sized pores, seeded with SCG cells. Cells were stained with calcein and thirty Confocal images were taken in row along the z-axis after 7 days in culture. Maximum projected images were generated with different color corresponding to different depth from the polymer surface. As shown in the color bar, pink is closest to the surface and red is at 150 µm from the surface. (c) SEM image of polymer (PLLA) scaffold without cells. (c) SEM image of a SCG cell cluster (indicated by arrow) inside a pore on day 2 after plating. (e): SEM image showing a neurite (indicated by arrow) from one cell to another on day 7 after plating. Bars represent 50 µm in (a), 100 µm (b), 50 µm in (c), 10 µm in (d) and 5 µm in (e).

Cell viability and spatial distribution was examined by calcein AM live cell staining, coupled with confocal microscopy, on day 7 after plating. Fig. 1a shows calcein stained SCG cells cultured on 2D flat surface. Fig. 1b shows the maximal projection of thirty images obtained by z-axis optical scanning and different colors were assigned according to the depth of the obtained image (color index shown as inset of fig. 1b). Cells were viable with well developed neurites in both 2D and 3D cultured conditions. In 3D scaffolds SCG cells intruded as deep as 150 µm from the top of the scaffold, which further confirmed that the pores inside the scaffolds were interconnected and had formed open channels to allow cell migration in all directions (Fig. 1b). SEM was further used to study the morphology of polymer scaffolds and 3D cultured SCG cells. As shown in Fig. 1c, polymer (PLLA) scaffolds have interconnected holes that can host cell for growth and multicellular aggregates formation. SEM images also showed that cells formed multi-cellular clusters inside the pores of the scaffolds (Fig. 1d) and had developed neurite connections between adjacent cells (Fig. 1e).

### 3D Cultured Cells Morphologically Resemble Cells from Freshly Dissected SCG Tissue

**Figure 2:**
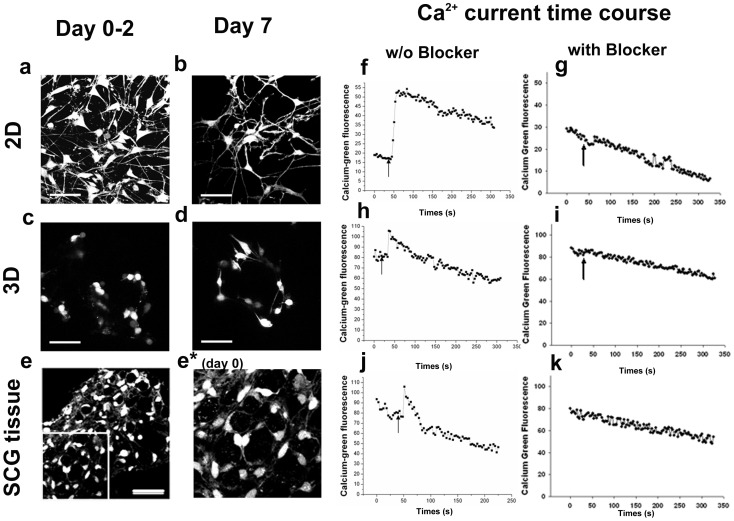
Cell morphology and high K^+^ depolarization induced intracellular calcium changes. Cell morphology was observed with a live cell indicator and cytoplasmic marker Calcein AM (a–e). Fluorescent images were captured by laser scanning confocal microscopy. (a) and (b) are confocal images of cells on 2D substrates on day 2 and day 7 after plating, respectively. (c) and (d) are volume rendered confocal depth projection images of cells on 3D PS scaffolds on day 2 and day 7 after plating, respectively. (e) is a volume rendered confocal image of the cells in a intact SCG tissue. (e*) is a blow-up of the insert in (e) to easily compare the morphology to that in (d). Bars represent 50 µm in all these 5 images. High K^+^ depolarization induced intracellular calcium changes were studied by Calcium Green, a calcium indicator (f–k). Intracellular calcium were reflected by Calcium Green’s fluorescent intensities and recorded by Confocal microscopy every 3 seconds. Shown in (f) and (h) are the typical calcium time course in responses to high K^+^ (50 mM) depolarization on 2D substrates and 3D PS scaffolds, respectively for day 2 cultures. (j) shows calcium time course from a typical responsive cell in an intact SCG tissue after dissection. (g), (i) and (k) are the typical calcium time course when calcium transient was suppressed by L-type calcium blocker from 2D, 3D and tissue samples, respectively. Arrows show the times points when high K^+^ buffer was added. The general decreasing trend of fluorescence intensity was resulting from photo-bleaching during recording.

Cell morphology was investigated with confocal images of calcein stained cells taken with higher magnification (60X) oil lens and quantitative cell morphology measurements were processed by SimplePCI 2000 software (Fig. 2a–e). Calcein is a cytoplasmic marker which gives a clear outline of intact cells thus can be utilized for cell morphological observation [22], [24]. Detailed results are presented in Tables 1 and 2. We used neurite density (the number of neurites per cell), neurite length, cell soma section area & roundness to characterize the cell morphology. Roundness, characterized by the circularity shape factor, calculated as: 4*Pi*area/perimeter^2^. Fig. 2a and 2b are SCG cells cultured on 2D surfaces, two and seven days after plating, respectively, while Fig. 2c and 2d are the same cells in 3D polymer scaffolds with same number of days in culture. As shown, on day 2 after plating, a few cells in 3D scaffolds had already developed short neurites and most cells were still round (Fig. 2c). Compared to the cells on 2D substrates (Fig. 2a), 3D cell neurite density was lower (0.7 vs. 2.4), and the neurites were shorter (10.9±3.2 µm vs. 38.9±17.7 µm, *p* = 7.88e−7). In comparison to that on 2D substrates, cells on 3D scaffolds spread poorly with smaller cell soma section area (90.0±22.3 µm^2^ v.s.149.8±59.3 µm^2^, *p* = 4.56e−6) and larger soma section roundness (0.81±0.05 vs. 0.51±0.11, p = 1.16e−15). In addition, a number of cells were found forming clusters which was not observed in 2D cells. On day 7 after plating, more neurites were observed among both 2D and 3D cells (Fig. 2b and 2d), with increased neurite lengths of 63.6±36.1 µm (*p* = 5.75e−10) and 25.2±13.8 µm (*p* = 0.001) respectively. The spreading condition of cells on 2D remained unchanged with comparable cell soma section area and roundness to the day 2 cells. However, the cells on 3D scaffolds were more spread with larger soma section area (144.5±56.9 µm^2^, *p* = 1.03e−4) and had lower roundness (0.64±0.15, *p* = 7.89e−6). The significant morphological difference between 2D and 3D cells observed on day 2 still existed among day 7 cells.

**Table 1:** Cell morphology on 2D substrates.

Days in culture	Neurite density	Neurite length	Soma section area	Soma section roundness
Day 2	121/50 = 2.4	38.9±17.7 µm (n = 121)	149.8±59.3 µm^2^ (n = 50)	0.51±0.11 (n = 50)
Day 7	88/11 = 8.0	63.6±36.1 µm (n = 88)*	134.5±72.1 µm^2^ (n = 11)	0.54±0.11 (n = 11)

* value was significantly different from that for Day 2 (p<0.05).

**Table 2:** Cell morphology on 3D PLLA substrates.

Days in culture	Neurite density	Neurite length	Soma section area	Soma section roundness
Day 2	11/16 = 0.7	10.9±3.2 µm (n = 11)#	90.0±22.3 µm^2^ (n = 16)#	0.81±0.05 (n = 16)#
Day 7	60/14 = 4.3	25.2±13.8 µm (n = 60)* #	144.5±56.9 µm^2^ (n = 14)*	0.64±0.15 (n = 14)* #

* value was significantly different from that for Day 2 (p<0.05).

# value was significantly different from that for 2D in Table 1.

Generally cells in 3D scaffolds developed shorter neurites and were less spread than the 2D cultured cells. The 3D cultured cell morphology (Fig. 2d) more closely mimicked the cell morphology found in freshly dissected intact SCG tissue (Figs. 2e and 2e*). The above differences between 2D versus 3D cultured cells suggested that polymer scaffolds can promotes cell attachment and differentiation that differs from what was observed with 2D substrates, which is consistent with conclusions from previous work [6], [7], [15], [25].

### VGCC Functionality

**Figure 3:**
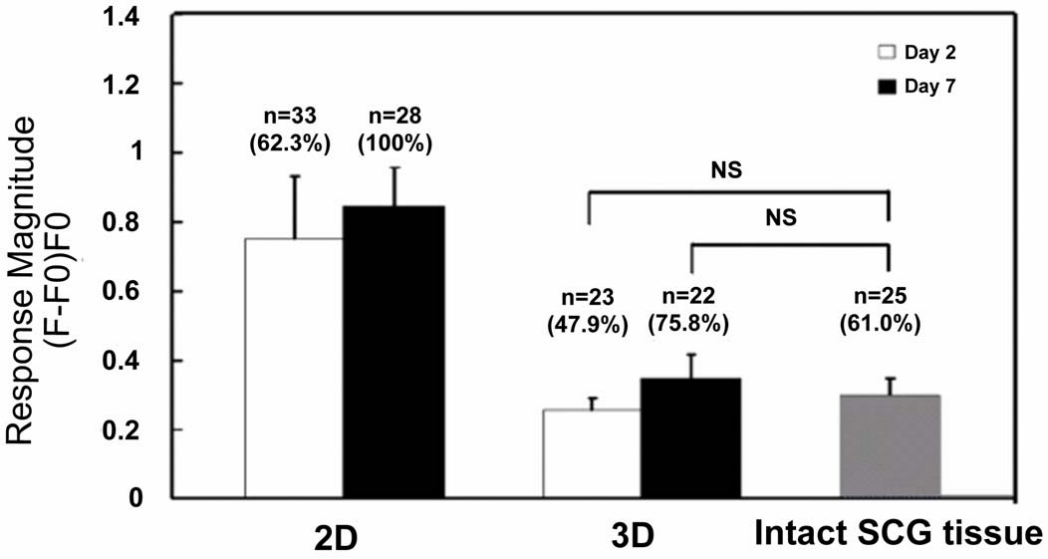
Cellular VGCC functionality. “NS” indicates that the means of the two samples compared are not significantly different with a level of p>0.8 by Student’s t-test. “n” is the number of responsive cells. The percentage of responsive cells from the total cell pool is indicated in parenthesis. 3D cells were cultured in PLLA scaffolds. Error bars are the 95% confidence intervals.

Previous studies done by our group and others have shown differences in calcium currents between intact and dissociated adult mouse SCG cells [26], and difference in VGCC function between 2D and 3D cultured human neuroblastoma cells or differentiated neural progenitor cells in collagen hydrogels, cytodex microbead scaffolds and polymer scaffolds [6], [7], [15], [25]. We reasoned that comparing VGCC functionality as reflected by calcium transient in response to high K^+^ (50 mM) depolarization is a convenient first step in confirming 3D and *in vivo* similarities. The time course intracellular calcium concentration was recorded as fluorescence of the membrane permeable dye Calcium Green-1 AM with a confocal laser scanning microscope. Fig. 2 (f–k) shows the typical time course changes in Calcium Green-1 AM fluorescence intensity under different conditions. Fig. 2f, 2h and 2j are responsive cells upon stimulation with high K^+^ on 2D substrates (f), 3D scaffolds (h) and intact SCG tissue (j). A cell was considered responsive only when it showed an increase in fluorescence intensity of 15% or higher over the basal fluorescence intensity level. The magnitudes of the response from each cell were expressed as a peak fractional increase over basal fluorescence intensity (F−Fo)/Fo, where F is the peak fluorescence intensity and Fo is the basal fluorescence intensity. The percentage and magnitude of cellular VGCC responses to high K^+^ depolarization within cells on 2D substrates, 3D scaffolds and intact SCG tissues are summarized in Fig. 3.

On day 2 after plating, 62.3% and 47.9% of cells on 2D substrates and 3D scaffolds respectively, were responsive to high K^+^ HBS buffer. The 3D cultured cells’ response magnitude was 0.26±0.08, which is much lower than the 2D cultured cells’ response magnitude of 0.75±0.54 (p = 0.0012). On day 7 after plating, the percentage of responsive 2D cultured cells increased to 100% as all the 28 cells measured had response to high K^+^ buffer, with the response magnitude increasing to 0.84±0.32, although the difference was not statistically significant (p = 0.43). The percentage of responsive cells on 3D scaffolds increased to 75.8% with the response magnitude increasing to 0.34±0.14 (p = 0.01). As with results on day 2, the response magnitude of cells on 3D scaffolds on day 7 was still significantly lower than that of cells on 2D substrates (*p* = 7.40e−7). It was interesting to observe that both the response magnitudes of 3D cells on day 2 and day 7 were not significantly different from that of cells in intact SCG tissue, which was 0.30±0.11 from 25 responsive cells from a pool of 41 cells (*p* = 0.88 and 0.80 respectively). Assuming that the cells in intact SCG tissue were not very different from cells *in vivo*, this observation provides evidence in support of the speculation that many cellular responses observed in 2D are probably exaggerations of *in vivo* function [27]. We are comfortable with the above assumption for several reasons. First, we made every effort to minimize the time between dissection and recording (below 60 min) and we maintained the tissue under physiological conditions (HEPES Buffer Saline with 3% Fetal Bovine Serum). Second, our approach is similar to investigations that utilize brain slices [28] and other peripheral nerve tissues [20], where *in vivo* similarity is well accepted.

It is well known that there are different types of VGCCs which are structurally homologous [29], [30]. Among those VGCCs, the dihydropyridine (DHP)-sensitive L-type channels and the currents caused by L-type VGCC have been thoroughly investigated and several related reviews have been published [31], [32], [33]. The high affinity of DHPs for the channels is a powerful test to differentiate L-type VGCC from non-L-type VGCC [34]. It has been shown that calcium transient induced by high potassium depolarization was mainly through L-type VGCC in neuronal cells [35], [36], [37]. We utilized a DPH class L-type VGCC blocker, Amlodipne besylate, to see if L-type VGCC was indeed the major player in our system. Amlodipne besylate is a well developed L-type calcium channel blocker that is marketed as an anti-hypertensive drug and for the treatment of angina. Amlodipne besylate has low cyto-toxicity; we have observed no cell death after treatment (5 µM for 90 minutes). Treated cells were depolarized with high K^+^ buffer and intracellular calcium concentration changes were followed with Calcium-Green florescence. After treated with the inhibitor, no cells were observed to be responsive, with little or no fluorescence increase observed (Fig. 2g, 2i and 2k), confirming that the calcium transient observed was indeed mainly due to L-type VGCC gating.

### VGCC mRNA Expression and Protein Localization

**Figure 4:**
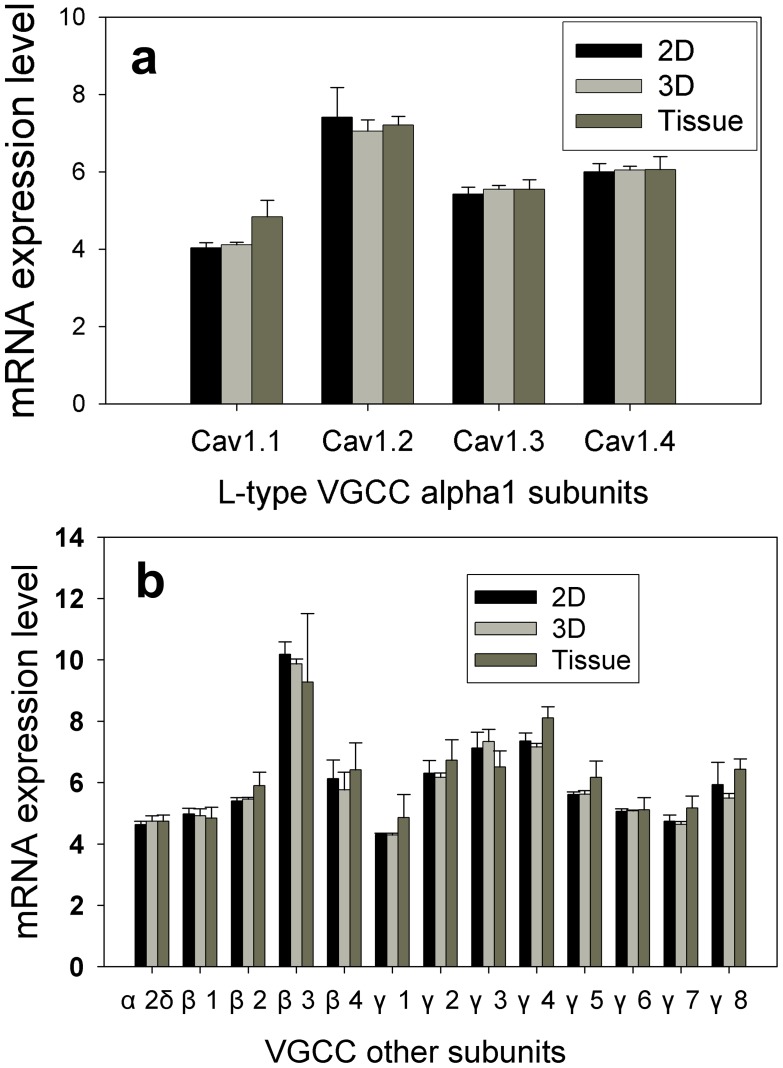
Microarray gene analysis results. Whole genome analysis was performed for SCG cells in 2D and 3D after 5 days in culture or in freshly dissected SCG tissue. Gene expression levels of L-type VGCC alpha 1 subunits (a) and other common subunits in VGCC (b) are presented here. The final gene expression levels were averaged from analysis of four biological replicates in each experimental condition (2D, 3D and tissue) (n = 4). Error bars represent the standard deviation. # Indicates the mean of expression level from tissue sample was significantly different from that of 2D or 3D cultures (in PS scaffolds) with p<0.05 by Student’s t-test.

To explore what could possibly be the reason of the lower calcium transient in 3D cultured cells and tissue, we examined the L-type calcium channel mRNA expression level with microarray gene chips. VGCC are formed as a complex from several different subunits: α_1_, α_2_δ, β_1–4_, and γ_1–8_. The α_1_ subunit forms the ion conducting pore while the associated subunits have several functions including modulation of gating. α_1_ subunit is also the major subunit that differentiates VGCC types. For L-type VGCC, there are four different α_1_ subunits, i.e. Ca_v_1.1 (*CACNA1S*), Ca_v_1.2 (*CACNA1C*), Ca_v_1.3 (*CACNA1D*) and Ca_v_1.4 (*CACNA1F*). Surprisingly we did not observe significant differences between 2D and 3D cultured SCG cells for all four α_1_ subunits (Fig. 4a). The mRNA levels for most of α_1_ subunits from SCG did not differ from the cultured cell samples either, with only Ca_v_1.1 (*CACNA1S*) slightly upregulated (significant at 0.05 level). Since L-type voltage gated calcium channel is composed of four different subunits, the expression of other subunits can be also very important in determining the expression of the whole channel. Thus we also examined the expression of α_2_δ (CACNA2D2), β (four family members β1 to β4 coded as CACNB1 to CACNB4) and γ subunits (eight members, γ1to γ8 coded as CACNG1 to CACNG8). Again, we did not observe any significant difference in mRNA expression for those subunits (Fig. 4b).

**Figure 5:**
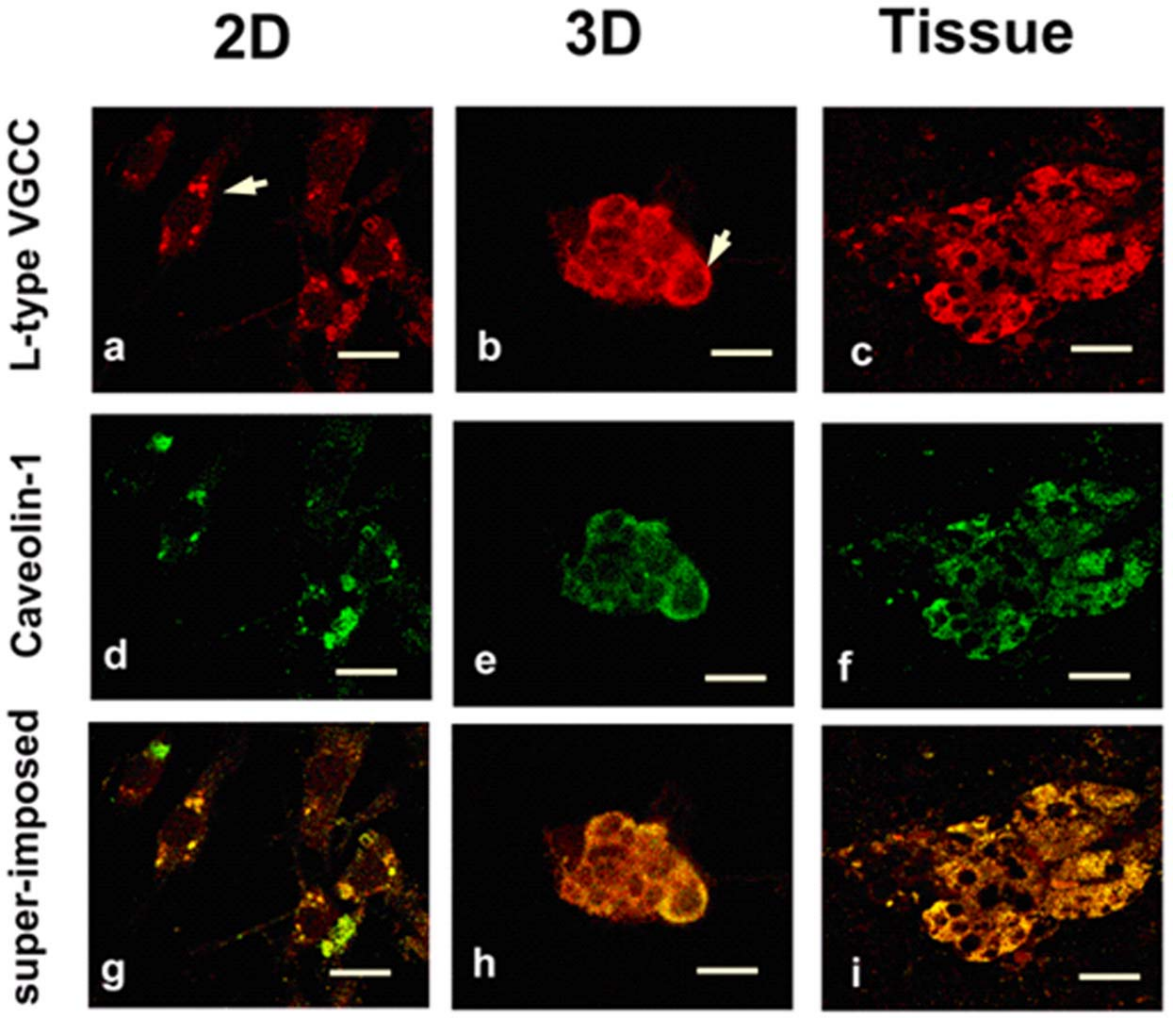
L-type VGCC and caveolin-1 colocalization. (a)–(c) Confocal images of L-type calcium channel staining of SCG cells cultured on 2D surface (a), 3D scaffolds (b) and in freshly dissected SCG tissue (c). Arrows point to one cell. (d)–(f) Confocal images of caveolin-1 (caveolar protein) from SCG cells cultured on 2D surface (d), 3D scaffolds (e) and in freshly dissected SCG tissue (f). (g)–(i) are the super-imposed image of VGCC and caveolin-1 staining, yellow indicates that VGCC protein and caveolin-1 are colocalized. Scale bars are 20 µm.

Although mRNA expression level of all L-type VGCC subunits were not different between 2D and 3D cultured cells, this finding could not rule out the possibility that the VGCC functionality differences are due to channel expression difference. To study if the voltage gated channel protein had been translated from mRNA and how the proteins are localized, we stained the α_1_ subunits of L-type VGCC (Fig. 5a–c) with antibodies. We observed that L-type calcium channel has distinctive localization in 2D cultured cells when compare to 3D and tissue sample. In 2D cultured SCG cells, L-type calcium channels are clustered together, forming bright punctuated morphology (Fig. 5a), while in 3D and tissue SCG cells, they are diffused all over the plasma membrane (Fig. 5b&c), suggesting differences in calcium channel signaling platform architecture.

### Cell Membrane Architecture

Ca^2+^ signaling proteins are known to be concentrated in lipid rafts/caveolar microdomains [38], [39], [40], [41], [42], which are dynamic flask shaped regions of cell membranes enriched with cholesterol and sphingolipid [43]. These microdomains appear to function as unique signal transduction platforms which tend to enrich certain signaling molecules while excluding others [43], [44], [45], [46]. Since lipid raft can concentrate signaling related proteins and facilitate the interaction, they provide a favorable environment for a number of signaling transductions [46]. More importantly, Ca^2+^ signaling has been shown to be initiated from lipid raft microdomains [38], in which Ca^2+^ channels and their regulatory proteins are clustered for regulation of Ca^2+^ mediated cellular function [46], [47], [48], [49], [50]. To examine whether in 2D cultured SCG cells, L-type calcium channels are concentrated more in lipid raft area that can facilitate calcium signaling, we double stained the cells with caveolin-1 antibody and L-type VGCC (Fig. 5a–f). Caveolin-1 is a caveolar protein and has been widely used as a caveolar lipid raft marker [51], [52]. The colocalization of L-type calcium channel and caveolin-1 was demonstrated by superimpose L-type VGCC channel confocal images over caveolin-1 confocal images (Fig. 5.g–i). The yellow color indicates the presence of both VGCC protein and caveolin-1. We observed that caveolin-1 spatial distribution was different between 2D and 3D cultures. In 2D cultured SCG cells, caveolin-1 had a punctuated distribution, while in 3D cultures and tissue it had a more diffused distribution. A similar differential protein spatial distribution pattern (punctuated versus diffused) between 2D and 3D samples has been observed with other proteins, e.g. focal adhesion kinase (FAK) [15], [53]. Integrins stimulate the activation extracellular signal-regulated kinase (ERK) through multiple signal transduction pathways. For example, ERK can be activated through caveolin and several other moieties. Another interesting observation was that caveolin-1 had a stronger signal at the edge of the microtissues formed in 3D scaffolds (Fig. 5e). From Fig. 5 g–i, almost all the red color in 2D samples are well superimposed with green, which indicating that almost all the VGCC proteins are located in caveolar lipid raft areas. However, in 3D and tissue samples some red color outside the green area is observable, indicating that some of the VGCC proteins are outside the caveolar lipid raft area. These results suggest that high calcium signals from 2D samples may result from VGCC proteins being well clustered in the lipid raft area in which they interact with other proteins that enhance their response to high K^+^ depolarization.

## Discussion

Scaffold curvature radius less than 100 µm compromises cell spreading and attachment along the bending direction and form the basis of contact guidance along the cylindrical substrata [25], [53]. In our case, the pore curvature radius falls into the range to produce topographical effects. Another important feature is the inter-connected pores, which can host cell clusters formed by the cells seeded into the same pore. Cells in multi-cellular organizations significantly differ from cells on flat 2D surfaces [54].

In this study, we found that the cell morphology and VGCC function from cells in 3D scaffolds more closely modeled intact SCG tissues in comparison to cells on 2D surfaces. Similar 3D effects have been observed in other 3D cell culture studies. Wang and Good reported that culturing PC 12 neuron–like cells and SHSY-5Y neuroblastoma cells in a rotating bioreactor resulted in formations of cell clusters and inhibition of neural extensions [55]. Wu and others observed a similar phenomenon from SHSY-5Y cells cultured on Cytodex 3 mircobead scaffolds [25]. Furthermore, Webb and others found changes in extra-cellular matrix (ECM)-related gene expression consistent with decreasing cell migration and increasing tissue formation when fibroblast cells were transferred from 2D to 3D culture on porous Tecoflex-derived biomaterials [56], which is similar to the polymer scaffolds we used in this study.

Intracellular calcium transient in response to high potassium depolarization was identical between 3D cultured and intact SCG tissue cells, but significantly different between 3D and 2D cultured cells. The intracellular calcium transient caused by high potassium depolarization was almost fully blocked by L-type voltage gated channel blocker. This is in contrast to previous findings where other non-L-type VGCCs were found to be expressed in SCG cells [57]–[69]. Among all the types of VGCCs, L-type VGCCs are known to couple depolarization of the plasma membrane to a wide range of cellular responses [60]. Ca^2+^ entry via L-type VGCCs triggers activation of the Ryanodine Receptor Type 2 (RyR_2_) in cardiac cells and initiates Ca^2+^-induced Ca^2+^-release [61], which greatly amplifies the cellular calcium transient required for effective initiation of contraction [60]. The similar association of L-type VGCCs and RyR_2_ was also found in rat hippocampus neurons [62]. By blocking L-type VGCCs, we blocked the amplification of cellular calcium transient through Ca^2+^-induced Ca^2+^-release. In neuronal cells, L-type VGCCs were found mainly in cell bodies and proximal dendrites [63], while other types of VGCCs such as P type and N type are more concentrated in nerve terminals and dendrites. In our experiments, we focused our observation on cell bodies only. This is a possible reason behind the observed little or no cellular calcium transient after L-type VGCCs inhibition.

Although mRNA of L-type VGCC subunits were not differently expressed by 3D and 2D cultured cells, immunostaining of L-type VGCC shows that the distribution of VGCC were different between 3D and 2D cultured cells but similar between 3D cultured cells and tissue samples. L-type VGCCs class C alpha subunit (CaV1.2) and class D alpha subunit (CaV1.3) were both found to be concentrated at the cell bodies and proximal dendrites in rat hippocampus and cerebral cortex neurons [63]. These two classes of subunit were observed to have very different distribution patterns, with class D more diffused and class C punctuated. Immunofluorescence staining of hippocampal sections of 2-month-old APP−/− and WT mice showed that CaV1.2 has a more diffused distribution pattern [64]. We speculated that the different pattern of calcium channel distribution may be related to function. Our results provide evidence in support of the hypothesis that some cellular responses under traditional 2D environment are exaggerated [27].

There are no published studies specifically designed to shed light on the mechanisms behind the “2D exaggeration hypothesis.” In terms of VGCC activity, the results presented here point to spatial channel distribution and more specifically punctuated distribution (2D) versus diffused (3D) pattern, which led us to suspect the presence of lipid rafts playing a role in exaggerating the 2D calcium signals. The parallel punctuated/diffuse distribution of caveolin-1 (caveolar lipid raft marker) was consistent with our speculation, pointing to fundamental differences in membrane architecture between 2D and 3D/tissue preparations.

From Fig. 5 (e), a higher caveolin-1 protein signal was observed on the edge of microtissues formed in 3D scaffolds. Cells at the edge of the microtissue are typically in contact with the polymer scaffold’s rigid surface. It is well known that cells in contact with rigid surfaces tend to adapt to the microenvironment and exhibit a more rigid architecture, measured in terms of higher Young’s modulus. One of the most abundant composition of caveolae lipid rafts are sphingolipids containing long, largely saturated acyl chains, which pack more tightly together [65], [66]. Thus lipid raft generally are more compact and rigid. In *in vivo* situation, all cells are in contact with each other and have lower Young’s modulus compared to 2D cells cultured on rigid surfaces. It is reasonable to suggest that 3D cultured cells model *in vivo* conditions better because, with the exception of cells on microtissue aggregate periphery in contact with rigid surfaces, the majority of cells inside the microtissue aggregate are in contact with neighboring soft cells as the case is *in vivo*.

Through use of freshly dissected SCG cells, we have shown that 3D cultured cells emulate the *in vivo* condition, with respect to VGCC function, better than 2D cultured cells. The explanation consistent with our findings is differences in membrane architecture. The practical issue that needs addressing is the meaning of our findings in the context of pre-clinical high throughput screening (HTS) of compounds with 2D versus 3D cell-based assays. In the design and validation of HTS assays, an assessment of the screening data, by measurements such as standard deviation (SD) or coefficient of variation (CV), is critical in determining whether an assay can identify hits with confidence. Zhang et al. [67] introduced the Z’ factor, a simple statistical dimensionless number [Z’ = (3σ_c+_+3σ_c−_)/(|µ_c+_−µ_c−_|)] that evaluates HTS assay quality with respect to identifying hits with a high degree of confidence. Where σ_c+_ and σ_c−_ denote signal standard deviations of positive and negative controls, respectively, and µ_c+_ and µ_c−_ denote signal means of the positive and negative controls, respectively.

Use of Z’ factor is now an accepted industry standard. If the Z’ factor is sufficiently large (>0) at the defined conditions, then the assay can be used in HTS. Typically, a Z’ factor equal or greater than 0.5 characterizes a robust assay for HTS. We have recently used a calcium assay in a 2D/3D comparative study of the type of scaffolds used in this study and a few commercially available 3D plates. As expected, 2D plates exhibited statistically higher Z’ factor (0.79), but all the 3D plates, including one from our laboratory, supported robust assays with all the Z’ factors higher than 0.5 [68]. These results confirmed the expectation that in comparison to 2D, 3D calcium assays are more likely to yield high σ_c+_ and lower µ_c+_ values, resulting in comparatively lower Z’ factors for 3D assays. If a physiologically more relevant 3D cell-based calcium assay exhibit a lower Z’ factor, the physiologically less relevant 2D cell-based calcium assay counterpart is more likely to yield false positive hits. Conversely, more physiologically relevant 3D calcium cell-based assay would be more effective in eliminating false positive hits and as such improve the overall yield from drug screening campaigns. Given that many drugs achieve their efficacy by interacting with membrane-integrated ion channels or their associated receptor-ligand behavior [69], this result brings attention to the potential importance of introducing three dimensional cell-based assays in drug discovery programs.

This study constitutes the first step toward comparing 3D cultures to their *in vivo* counterparts within the cell-based assay HTS context. The results support the following conclusions.

1. Neonatal mouse SCG cells cultured in 3D PLLA or PS scaffolds more closely mimic the cells in intact SCG tissue than those cultures on 2D substrates with respect to high K^+^-mediated calcium transient.
2. The exaggerated VGCC function from 2D cultured SCG cells may partly be explained by differences in membrane architecture, characterized by uniquely organized caveolar lipid rafts.
